# Editorial: Artificial intelligence and social robotics for mental healthcare

**DOI:** 10.3389/frobt.2025.1665800

**Published:** 2025-09-23

**Authors:** José-Antonio Cervantes, Luis-Felipe Rodriguez, J. Octavio Gutierrez-Garcia, Francisco Cervantes-Alvarez, Miguel Vargas Martin, Sandra Baldassarri

**Affiliations:** 1 Department of Computer Science and Engineering, Universidad de Guadalajara, Ameca, Mexico; 2 Department of Computing and Design, Instituto Tecnologico de Sonora, Ciudad Obregon, Mexico; 3 Department of Computer Science, ITAM, Mexico City, Mexico; 4 Department of Electronics, Systems and Informatics, ITESO - Jesuit University of Guadalajara, Tlaquepaque, Mexico; 5 Faculty of Business and Information Technology, Ontario Tech University, Oshawa, ON, Canada; 6 Engineering Research Institute of Aragon (I3A), Computer Science Department, Universidad de Zaragoza, Zaragoza, Spain

**Keywords:** social robotics, mental healthcare, ethical considerations, human-robot interaction, artificial intelligence

## Introduction

In recent decades, mental health disorders such as anxiety and depression have risen dramatically across the globe (Baker and Kirk-Wade, 2023; Ten Have et al., 2023). Artificial Intelligence (AI) and Social Robotics present groundbreaking prospects that could revolutionize mental healthcare (Hung et al.). Social robots, specifically designed for human interaction by displaying emotions and engaging in conversations, along with data-driven AI, exemplify modern advancements in sectors such as education (LeTendre and Gray, 2024) and elderly care (Yen et al., 2024). Despite these advancements, the convergence of AI and social robotics in mental health has untapped potential and presents a plethora of unresolved challenges. For instance, there is a lack of ethical norms (Torras, 2024) that regulate and guide the correct use of social robots in a wide variety of contexts, ranging from assistive care for the elderly to healthcare. Therefore, in this Research Topic, we have collected current contributions to the design and development of social robots as well as empirical studies on human-robot interactions focused on mental health and elderly care.

## Articles of the Research Topic

The articles contributing to the design and evaluation of social robots (supported by artificial intelligence) for mental healthcare included in this Research Topic are as follows:

Aoki et al. focused on how different forms of humanoid robot vitality (namely, gentle and rude) impact human performance with an emphasis on mental workload. Particularly, their study explored human performance and emotional states while engaging in demanding cognitive multitasking in the presence of social robots. Experiments involving 29 participants were conducted with an iCub humanoid robot continuously displaying vitality forms through coordinated movements of its arms, torso, and head. The participants interacted with the iCub robot while performing a task battery simulating cognitive challenges encountered by aircraft pilots. Whereas some participants interacted with iCub exhibiting a rude vitality form, the others interacted with iCube exhibiting a gentle vitality form. While interacting with the robot, Aoki et al. recorded participants’ facial expressions and electrodermal activity to assess their mental workload. Their results revealed that a robot exhibiting a gentle vitality form fostered a more positive and lower mental workload than one exhibiting a rude vitality form. Aoki et al. results offer useful insights into stress-free human-robot interaction supporting mental wellbeing.

Hung et al. present an empirical study involving 46 elderly participants (from 60 to over 100 years old) on the use of social robots in the mental health area. Their study aimed to identify ethical challenges and propose mitigation strategies for implementing social robots in long-term care settings. To achieve these objectives, their research involved human-robot interaction between elderly individuals and social robots (namely, Paro and Lovo robots) in separate studies. As a result of this empirical study, four key ethical challenges associated with the implementation of social robots in long-term care facilities were identified: inequitable access, participant consent, human care substitution, and concerns about infantilization. In addition, their work discussed mitigation strategies to address ethical support for older adults in long-term care settings.

Dong et al. explored children’s understanding of social robots endowed with artificial intelligence capabilities and how social robots promote learning engagement within the context of science, technology, engineering, arts, and mathematics (STEAM) education. Motivated by a lack of research on the effectiveness of social robots into primary and secondary education in informal settings, their objective was to design and evaluate a theater afterschool program (supported by social robots) to promote STEAM education and foster embodied learning. Their study took place in an elementary school involving 38 children. The children interacted with different robots, ranging from humanoid robots with human-like facial expressions to stereotypical robots. Among the participants, there were children with autism spectrum disorder, which (according to Dong et al. results) improved their social and emotional skills by interacting with social robots.

Liu et al. present an empirical study on the influence of behaviorally anthropomorphic service robots on customer variety-seeking behavior. Their study aimed to identify the mechanisms through which robot anthropomorphism affects consumer choices, specifically examining social presence as a mediator and decision-making context as a moderator. In pursuit of these goals, their research involved a series of six experiments with ordinary consumers interacting with service robot scenarios. They found that higher behavioral anthropomorphism significantly increases variety-seeking, a relationship partially mediated by social presence. Additionally, the influence of social presence on variety-seeking was significantly stronger in public decision-making contexts. Their work provides recommendations for strategically deploying service robots to enhance customer engagement. Liu et al. results have implications related to mental healthcare because social, anthropomorphic robots should deliver services (e.g., psychological therapies and counseling), expressing emotions (as expected by users) in order to establish both a social and an emotional connection.

Kamide et al. carried out a psychological evaluation of an avatar robot in two distinct regions (Dubai and Japan) to explore how cultural background influences psychological aspects attributed to robot avatars. Furthermore, aimed at understanding whether (avatar) robots’ fundamental psychological impressions such as warmth, competence, and discomfort are essential for developing more culturally adapted human-robot interactions. Specifically, they conducted two studies: the first involved a virtual robot used as an avatar, and the second involved a physical robot. The results suggest that participants from Dubai were more comfortable interacting with a virtual robot, whereas participants from Japan were more comfortable interacting with a physical robot. Kamide et al. also discussed the implications of these findings and the relationship between robot evaluations and cultural background, which is a relevant aspect for social robots designed for mental healthcare in multicultural domains.

We believe this collection of articles offers valuable insights supported by empirical evidence to further advance in social robots for mental healthcare. We hope this Research Topic motivates the readers to embark on and contribute to this fascinating research area.
